# Neurocomputational mechanisms of food and physical activity decision-making in male adolescents

**DOI:** 10.1038/s41598-023-32823-x

**Published:** 2023-04-15

**Authors:** Seung-Lark Lim, Amanda S. Bruce, Robin P. Shook

**Affiliations:** 1 Department of Psychology, University of Missouri-Kansas City, 5030 Cherry St, Kansas City, MO 64110 USA; 2 Center for Children’s Healthy Lifestyles & Nutrition, Department of Pediatrics, Children’s Mercy, 610 E. 2nd St, Kansas City, MO 66108 USA; 3 Department of Pediatrics, University of Kansas Medical Center, 3901 Rainbow Blvd, Kansas City, KS 66160 USA; 4 School of Medicine, University of Missouri-Kansas City, 2411 Holmes, Kansas City, MO 64108 USA

**Keywords:** Neuroscience, Psychology

## Abstract

We examined the neurocomputational mechanisms in which male adolescents make food and physical activity decisions and how those processes are influenced by body weight and physical activity levels. After physical activity and dietary assessments, thirty-eight males ages 14–18 completed the behavioral rating and fMRI decision tasks for food and physical activity items. The food and physical activity self-control decisions were significantly correlated with each other. In both, taste- or enjoyment-oriented processes were negatively associated with successful self-control decisions, while health-oriented processes were positively associated. The correlation between taste/enjoyment and healthy attribute ratings predicted actual laboratory food intake and physical activities (2-week activity monitoring). fMRI data showed the decision values of both food and activity are encoded in the ventromedial prefrontal cortex, suggesting both decisions share common reward value-related circuits at the time of choice. Compared to the group with overweight/obese, the group with normal weight showed stronger brain activations in the cognitive control, multisensory integration, and motor control regions during physical activity decisions. For both food and physical activity, self-controlled decisions utilize similar computational and neurobiological mechanisms, which may provide insights into how to promote healthy food and physical activity decisions.

## Introduction

### Energy balance and the role of the brain

Body weight is regulated by energy intake and expenditure. While what we eat and drink determines energy intake, what we do (physical activity) and the resting metabolic rate (RMR) determine energy expenditure. At the most basic level, a positive energy balance (energy intake > energy expenditure) causes weight gain, and a negative energy balance (energy intake < energy expenditure) causes weight loss. Thus, to prevent obesity risks and promote a healthy lifestyle, a mechanistic understanding of the psychological and neurobiological control processes for energy intake and energy expenditure that maintain a healthy and stable energy balance is required. The brain plays a critical role in the regulation of energy homeostasis (intake and expenditure) and metabolism^1–4^. The brain monitors and detects body excess or deficit by integrating multiple metabolic information (e.g., nutrients, gut-driven satiety signals, and adiposity-related hormones). The regulatory body metabolic mechanisms that the brain controls include food-seeking behavior, gastric emptying, nutrient uptake in the gut, thermogenesis, hepatic glucose production, insulin secretion, and glucose/fatty acid metabolism in adipose tissue and skeletal muscle. Recently, decision neuroscience has significantly contributed to our understanding of computational and neurobiological mechanisms of food decision-making (energy intake)^5–7^, but this is not the case for physical activity decision-making (energy expenditure)^8^. Considering the interdependent nature of food and physical activity decisions in the control of healthy energy balance, it is critical to advance our scientific understanding of decision-making processes for both energy intake and expenditure. An intervention strategy that targets only one aspect (energy intake or expenditure) would be insufficient or less effective^9^.

### Self-controlled food and physical activity decisions

To make healthy (or self-controlled) food decisions (e.g., an apple over fries), we must prioritize the healthiness of foods that provides long-term nutritional benefits over delicious taste that produces short-term pleasurable experience. Similarly, to make healthy (or self-controlled) physical activity decisions (e.g., jogging over playing a video game), we must emphasize the healthiness of activities that provides long-term physical benefits, rather than an enjoyable experience that produces short-term pleasure or comfort. However, it should be noted that the healthiness of given food or activity is dependent on the individual’s various health and contextual factors (e.g., milk products for people with lactose intolerance and running for patients with cachexia). In general, voluntary healthy energy intake and expenditure decisions will require effortful and goal-directed self-control operations that share interlinked executive functions such as cognitive control, inhibition, and future thinking^10^. Yet, the brain’s executive functioning does not reach full maturity until young adulthood^11–13^, which makes adolescents prone to impulsive and immediate reward-oriented decisions rather than deliberate future goal-oriented decisions^12^. Furthermore, as well as the neural plasticity of the developing brain, adolescence is the critical period for life-long health habit formation^14^ and the critical period of nutrition needs for pubertal growth^15^. During adolescence, the development of the brain, body, and self-control behaviors tightly interact with each other, which all contribute to energy balance regulation. The body composition, behavioral habits, and preferences attained during adolescent periods often tack into adulthood^16^. Thus, a scientific understanding of adolescents’ decision-making processes for food and physical activity is a requisite for effectively guiding them to establish decision-making patterns or habits for healthier energy balance.

### The current study

This pilot neuroimaging study investigated how male adolescents make food and physical activity decisions, and how these decisions are influenced by body weight status and physical activity levels. To accomplish this, we used behavioral tests and functional magnetic resonance imaging (fMRI). We hypothesized that (1) physical activity decisions (energy expenditure) share similar neurocomputational mechanisms with food decisions (energy intake), and (2) body weight status and physical activity levels modulate neurocomputational processes of food and physical activity decisions. Particularly, we hypothesized that adolescent males’ food and physical activity decisions would be determined by how participants evaluate and incorporate the immediate reward-related attribute (i.e., the tastiness of foods; the enjoyment of physical activities) and the future-oriented reward attribute (i.e., the healthiness of foods and the healthiness of physical activities) into their decision-making process (see Eqs. 1 and 2). Also, we hypothesized that these participants’ decision weights for the immediate reward-related and future-oriented reward attributes would predict self-controlled food and physical activity decisions. Based on previous neuroimaging literature^17–20^, we hypothesized the ventromedial prefrontal cortex (vmPFC) would encode participants’ decision values for both foods and physical activities at the time of choice, which was tested by model-based fMRI data analyses^21,22^.

1 $$Food\, Decisions= {\beta }_{1}\,\times\, Food \,Taste \,Ratings\, + \,{\beta }_{2}\,\times\, Food \,Health\, Ratings\, + \,\epsilon$$

2 $$Physical \,Activity\, Decisions\,= \,{\beta }_{1}\,\times \,Activity\, Enjoyment\, Ratings \,+ \,{\beta }_{2}\,\times \,Acitivy \,Health\, Ratings\, + \,\epsilon .$$

## Results

### Behavioral results

Descriptive statistics of behavioral ratings for thirty-eight adolescent males (14–18 years old; mean 15.89) are reported in Table S1. For rating data, we examined how subjective taste/enjoyment and health attribute ratings are related for 60 food and 60 physical activity items, respectively. For each individual, we first calculated Pearson correlation coefficients between two attribute ratings separately for food and activity items (Fig. 2A). Then, we performed one-sample *t* tests (against zero) with estimated correlation coefficients for group-level analyses. The correlation coefficients between taste and health attribute ratings for foods were widely distributed across participants, which was not significant at the average group level, *mean r* = −0.02, *SD* = 0.27, *t*_(37)_ =  −0.52, *p* = 0.605,* d* = −0.09. On the other hand, the correlation coefficients between enjoyment and health attribute ratings for physical activities were significant at the average group level, *mean r* = 0.20, *SD* = 0.22, *t*_(37)_ = 5.47, *p* < 0.001,* d* = 0.90. The correlation between food attribute correlation coefficients, *r*_(taste, health)_, and physical activity attribute correlation coefficients,* r*_(enjoyment, health)_, was not significant, *r*_(36)_ = 0.29, *p* = 0.079. None of the correlation coefficients between taste/enjoyment and health ratings showed a significant correlation with age (all *p* values > 0.05). To determine whether these correlations between taste/enjoyment and healthy attributes vary by body weight status (NW, OW/OB) and physical activity (ACT, SED) levels, we conducted 2 by 2 ANOVAs. The ANOVA on the food taste and health rating correlations did not show significant results, all *p* values > 0.05. But the ANOVA on the physical activity enjoyment and health rating correlations showed a significant main effect of the physical activity level, *F*_(1,34)_ = 6.11, *p* = 0.019, *η*^2^_*p*_ = 0.15, indicating the ACT group demonstrates higher correlations between enjoyment and health ratings compared to the SED group (Fig. 2B). The other effects were not significant, all *p* values > 0.05.

### Computational models of taste/enjoyment and health attribute integration

**Figure 1:**
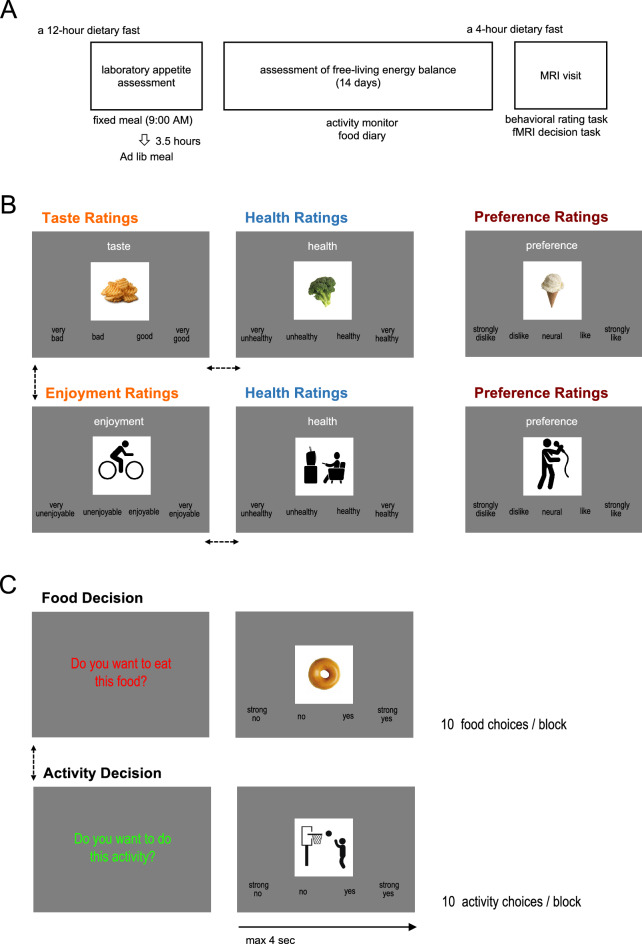
**(A)** Overview of the study design. (**B**) Adolescents completed food and activity ratings before fMRI scans. They provided taste and health attributes and overall preference ratings for 60 different food items, and enjoyment and health attributes and overall preference ratings for 60 different activity items. The order of food and activity rating tasks and the order of attribute ratings within each type of task were randomized. (**C**) fMRI decision task consisted of activity and food decision blocks. Participants completed 6 runs of the decision task and each run included 3 food and 3 physical activity choice blocks (10 decision trials per block). Computer algorithms randomized the order of blocks and the order of trials within the block. For each food and physical activity image, participants entered their decisions using a 4-point scale (“strong no–strong yes” or “strong yes–strong no”; counterbalanced across participants) within 4-s.

**Figure 2:**
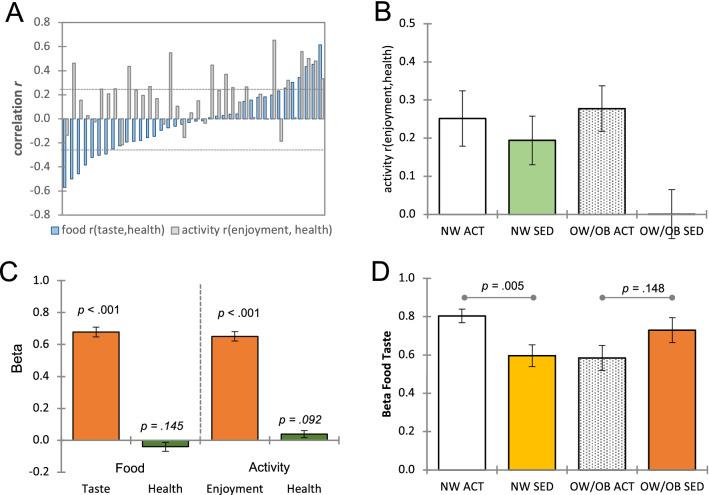
(**A**) Participants’ correlation coefficients of food taste and health ratings and activity enjoyment and health ratings are shown in ascending order of food correlation coefficients. The dotted lines indicate the critical *r* values at the individual level (± 0.254 at *p* < 0.05). (**B**) The correlations between activity enjoyment and health ratings were significantly different between active (ACT) and sedentary (SED) groups. (**C**) Adolescents’ food decisions were solely predicted by taste ratings, and activity decisions were solely predicted by enjoyment ratings. (**D**) The food taste beta weights were significantly different between normal weight active (NW ACT) and normal weight sedentary (NW SED) groups, while they were not different between overweight/obese active (OW/OB ACT) and overweight/obese sedentary (OW/OB SED) groups. All error bars denote standard errors. *n* = 38.

To test our multi-attribute computational model (see Eqs. 1 and 2), we examined how participants incorporate taste and health attribute values into their food decisions, and enjoyment and health attribute values into their physical activity decisions. For each individual, we first fitted linear regression models of taste/enjoyment and health ratings (measured by 4-point scales during the behavioral rating task; Fig. 1B) on participants’ decisions (measured by a 4-point scale during the fMRI decision task; Fig. 1C) separately for food and physical activity items. Then, for group-level analyses, we conducted one-sample *t* tests (against zero) with the estimated regression coefficients (= decision weights). For food items, taste ratings, *mean β* = 0.68, *SD* = 0.19, *t*_(37)_ = 21.47, *p* < 0.001, *d* = 3.57, but not health ratings, *mean β* = -0.04, *SD* = 0.17, *t*_(37)_ =  −1.49, *p* = 0.145,* d* = −0.24, significantly predicted participants’ food decisions. Similarly, for physical activity items, enjoyment ratings, *mean β* = 0.65, *SD* = 0.03, *t*_(37)_ = 20.78, *p* < 0.001, *d* = 3.45, but not health ratings, *mean β* = 0.04, *SD* = 0.02, *t*_(37)_ = 1.63, *p* = 0.092, *d* = 0.28, significantly predicted participants’ physical activity decisions (Fig. 2C). These results suggest that, on average, male adolescents *do not* incorporate health information into their food and physical activity choices (i.e., pleasure-oriented decision processes), despite possessing the knowledge of health values (as demonstrated by significant health rating differences between healthy and unhealthy food items,* t*_(37)_ = 24.55, *p* < 0.001,* d* = 3.98, as well as between active and sedentary physical activity items, *t*_(37)_ = 19.43, *p* < 0.001,* d* = 3.15). None of the taste/enjoyment and health beta-weights revealed a significant correlation with age, all *p* values > 0.05. To check whether these decision weights of taste/enjoyment and healthy attributes vary by body weight status (NW, OW/OB) and physical activity (ACT, SED) levels, we performed 2 by 2 ANOVAs. The ANOVA result on the beta weights of taste ratings on food decisions showed a significant interaction effect of body weight status and activity level, *F*_(1,34)_ = 9.82, *p* = 0.004, *η*^2^_*p*_ = 0.22 (Fig. 2D). None of the main effects was significant, all *p* values > 0.05. In simple analyses, the NW ACT group showed significantly higher taste beta weights compared to the NW SED group, *t*_(19)_ = 3.15, *p* = 0.005, *d* = 1.38, while the OW/OB ACT and OW/OB SED groups did not show a significant difference, *t*_(15)_ =  −1.52, *p* = 0.148, *d* = −0.75, suggesting that the food decision process in which adolescents process taste attributes can be systematically varied by their weight status and physical activity level. Quite interestingly, while the NW ACT male adolescents belong to a relatively healthy category, they still possess strong taste-oriented food decision processes which may serve as a potential health risk if they become no longer active. The 2 by 2 ANOVA results on the other 3 beta-weights showed no significant effect, all *p* values > 0.05.

### Relation between food and physical activity decisions

**Figure 3:**
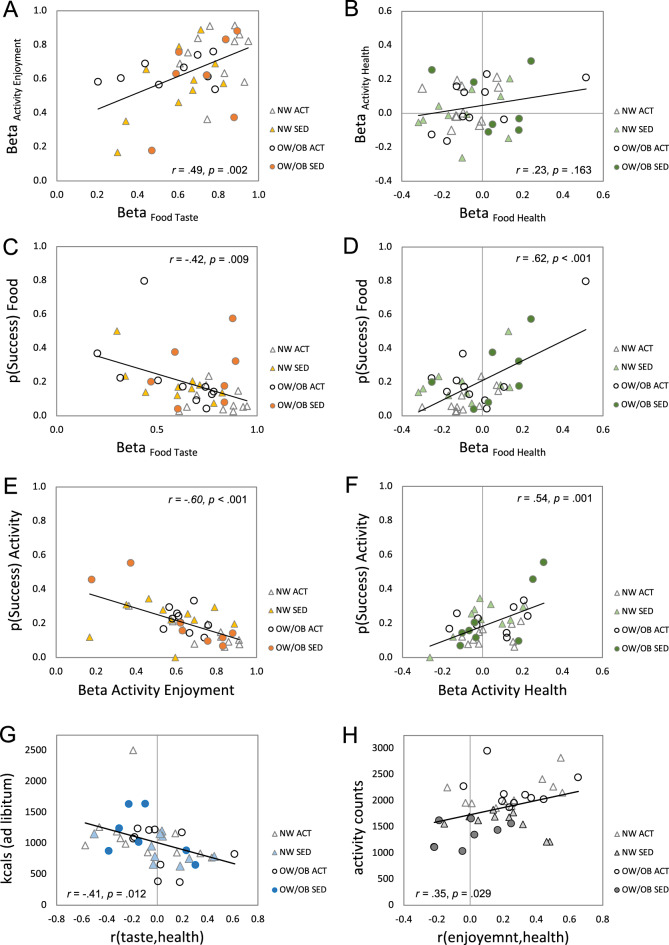
(**A**) The beta weights of food taste attribute were positively correlated with the beta weights of activity enjoyment attribute. (**B**) The beta weights of food health attribute were not significantly correlated with the beta weights of activity health attribute. (**C**) The beta weights of food taste attribute were negatively correlated with the proportions of successful self-control in food decisions. (**D**) The beta weights of food health attribute were positively correlated with the proportions of successful self-control in food decisions. (**E**) The beta weights of activity enjoyment attribute were negatively correlated with the proportions of successful self-control in activity decisions. (**F**) The beta weights of activity health attribute were positively correlated with the proportions of successful self-control in activity decisions. (**G**) The correlations between food taste and health attribute ratings were negatively associated with the amount of food consumption (kcals) at the ad libitum pizza buffet (*M* = 1023, *SD* = 369). (**H**) The correlations between physical activity enjoyment and health attribute ratings were positively associated with the physical activity level monitor measures of the 2-week assessment period (*M* = 1866, *SD* = 418). *n* = 38.

Next, to explore how the decision processes of food and physical activity are related to each other, we performed correlational analyses between the decision weights of food taste attribute and physical activity enjoyment attribute (both represent pleasure-oriented decision processes) as well as between the decision weights of food health attribute and physical activity health attribute (both represent health-oriented decision processes). The regression beta weights of food taste attribute and physical activity enjoyment attribute showed a significant positive correlation, *r*_(36)_ = 0.49, *p* = 0.002 (Fig. 3A), suggesting that participants who make stronger taste-oriented food decisions have a similar tendency to make stronger enjoyment-oriented physical activity decisions. However, the regression beta weights of food health attribute and physical activity health attribute were not significantly correlated with each other, *r*_(36)_ = 0.23, *p* = 0.163 (Fig. 3B).

### The role of self-control

By using choices from the fMRI decision task, we examined participants’ self-control execution on food and physical activity items. For self-control analyses^17^, we selected the self-control challenge trials based on participants’ subjective attribute ratings for 60 food and 60 activity items. For foods, ‘tasty but unhealthy’ items and ‘not tasty but healthy’ items were selected first, and then we calculated the proportion of successful self-control choices among them (‘strong no’ or ‘no’ responses for the tasty but unhealthy foods and ‘yes’ or ‘strong yes’ responses for the not tasty but healthy foods). Similarly, for physical activities, ‘enjoyable but unhealthy’ items and ‘not enjoyable but healthy’ items were selected, and then the proportion of successful self-control choices (‘strong no’ or ‘no’ responses for the enjoyable but unhealthy activities and ‘yes’ or ‘strong yes’ responses for the not enjoyable but healthy activities) were calculated. The means of the proportion of successful self-control food and physical activity choices were 0.18 (*SD* = 0.16) and 0.20 (*SD* = 0.11), respectively. Neither successful food nor physical activity self-control proportion correlated with age, all *p* values *p* > 0.05. It is worth noting there was a significant correlation between two successful self-control proportions, *r*_(36)_ = 0.41, *p* = 0.011, suggesting shared self-control mechanisms between food and activity choices. To check whether the proportions of successful self-control choices vary by body weight status and physical activity levels, we conducted 2 by 2 ANOVAs. But they did not show significant results, all *p* values > 0.05.

To further test whether our key variables (taste/enjoyment and health decision weights; correlations between attribute ratings) of computational models predict successful self-control in food and physical activity decisions, we performed a series of correlational analyses. For food choices, the beta weights of taste and health attributes revealed significant negative, *r*_(36)_ = −0.42, *p* = 0.009 (Fig. 3C), and positive correlations, *r*_(36)_ = 0.62, *p* < 0.001 (Fig. 3D) with the proportion of successful self-control decisions. Also, the correlation coefficients between food taste and health attribute ratings (see Fig. 2A) showed a significant positive correlation with the successful self-control food choices, *r*_(36)_ = 0.43, *p* = 0.007. In the multiple regression analysis in which three key decision model variables were entered simultaneously, *F*_(3,34)_ = 22.88, *p* < 0.001, *R*^2^ = 0.67, both taste and health attribute beta weights were significant, *β* = −0.53, *t*_(34)_ = −5.21, *p* < 0.001; *β* = 0.67, *t*_(34)_ = 5.77, *p* < 0.001, while the correlation coefficient between food taste and health attribute ratings was not, *β* = 0.08, *t*_(34)_ = 0.68, *p* = 0.499. Similarly, for physical activity choices, the beta weights of enjoyment attribute and health attribute revealed significant negative, *r*_(36)_ = −0.60, *p* < 0.001 (Fig. 3E), and positive correlations, *r*_(36)_ = 0.54, *p* = 0.001 (Fig. 3F) with the proportion of successful self-control decisions. However, the correlation coefficients between activity enjoyment and health attribute ratings (see Fig. 2A) did not show a significant correlation, *r*_(36)_ = 0.07, *p* = 0.679. In the multiple regression analysis, *F*_(3,34)_ = 15.72, *p* < 0.001, *R*^2^ = 0.58, both enjoyment and health attribute beta weights were significant, *β* = −0.53, *t*_(34)_ = −4.68, *p* < 0.001; *β* = 0.50, *t*_(34)_ = 4.11, *p* < 0.001, while the correlation coefficients between activity enjoyment and health attribute ratings were not significant, *β* = −0.04, *t*_(34)_ = −0.68, *p* = 0.504.

### Real-world implications

Lastly, to determine how our key model variables of food and physical activity decisions are linked to ‘actual’ food consumption and physical activity measures, we performed correlation analyses with the dietary assessment (average kcals/day from 2-week food diary; kcals consumed from ad libitum buffet) and the physical activity assessment (2-week VM average counts of activity monitor) data. For food decision model variables, the correlation coefficients between food taste and health attribute ratings, *r*_(taste, health),_ were negatively associated with the amount of food consumption from the ad libitum cheese pizza buffet, *r*_(36)_ = −0.41, *p* = 0.012, demonstrating that the participants who perceived unhealthy foods as tasty consumed more at the ad libitum buffet (Fig. 3G). Also, the regression beta weights of food taste attribute showed a marginal correlation value with the average energy intake measured from the 2-week food diary assessment (2 missing data), *r*_(34)_ = 0.30, *p* = 0.071. For physical activity decision model variables, the correlation coefficients between activity enjoyment and health attribute ratings, *r*_(enjoyment, health)_, were positively associated with the physical activity monitor measure, *r*_(36)_ = 0.35, *p* = 0.029, demonstrating that participants who perceived healthy physical activities as enjoyable made more physical activities during our 2-week assessment period (Fig. 3H). The regression beta weights of activity enjoyment and health attribute showed no significant correlation with the physical activity levels, all *p* values > 0.05.

### fMRI results

**Figure 4:**
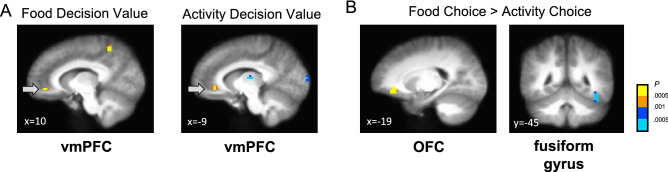
(**A**) Both food and physical activity-related decision values were positively correlated with vmPFC activities. (**B**) OFC showed stronger activity during food choices compared to physical activity choices, while fusiform gyrus showed stronger activity during physical activity choices compared to food choices. All images are threshold at *p* < 0.05 corrected with cluster size correction.

We examined fMRI data to identify the brain areas that parametrically encode food and physical activity-related variables representing participants’ decision values (i.e., how much they want to eat or want to do; ‘strong no’–‘strong yes’). Consistent with our decision model of food and physical activity, when participants made their choices, brain activation in the vmPFC positively correlated with subjective decision values in both conditions (*p* < 0.05 corrected; Fig. 4A; Table S2). Our findings firstly demonstrate that the physical activity decisions share similar neurocomputational circuitry operations with other types of rewards or commodities shown in other studies, like monetary choices^23,24^. Also, we examined two event indicator regressors to check potential task-related differences (e.g., motivational or cognitive demands) between food and physical activity choices. The orbitofrontal cortex (OFC) showed stronger activations during food choices compared to physical activity choices, while the fusiform gyrus showed stronger activations during physical activity choices compared to food choices (*p* < 0.05 corrected; Fig. 4B; Table S3). This finding suggests the potential task differences such as enhanced gustatory sensory processes during food choices and higher-order visual stimulus processes during physical activity choices.

**Figure 5:**
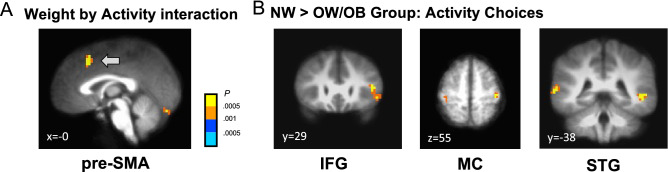
(**A**) Weight by activity group interaction effect was found in the pre-SMA. (**B**) Normal weight group (*n* = 21) compared to overweight/obesity group (*n* = 17) showed stronger IFG, motor cortex, and STG activations during activity choices. The images are threshold at *p* < 0.05 corrected with cluster size correction.

Next, to check whether the brain activities vary by the type of decisions (food, physical activity), body weight status (NW, OW/OB), and physical activity (ACT, SED) levels, we conducted an exploratory 2 by 2 by 2 repeated-measures ANOVA. A significant 2-way interaction effect by body weight status and physical activity levels was found in the pre-supplementary motor area (pre-SMA) (*p* < 0.05 corrected; Fig. 5A; Table S4), a brain area known to represent action intentions^25^ and prospective effort-related costs^26^. Subsequent analyses showed that the NW group showed stronger activations in the inferior frontal cortex (IFG), motor cortex, and superior temporal gyrus (STG) compared to the OW/OB group during physical activity choices (*p* < 0.05 corrected; Fig. 5B; Table S4). Contrarily, no clear group difference by physical activity levels was observed (Table S4).

## Discussion

This first-of-a-kind study investigated the neurocomputational mechanisms of food (energy intake) and physical activity (energy expenditure) decision-making and demonstrated that two decision processes are interdependent, not independent. More specifically, as described in our computational models (Eqs. 1 and 2), we hypothesized that (1) how participants subjectively evaluate two key attributes of foods (taste and health) and physical activities (enjoyment and health) and (2) how they incorporate those attributes into their decision process would determine their food and physical activity decisions. Not surprisingly, among participants, the associations between taste and health ratings of foods varied widely from strong negative to strong positive correlations (Fig. 2A). Our previous study in a younger sample suggested the negative association between healthiness and tastiness of foods (i.e., unhealthy foods taste better) is one of the underlying susceptibility factors to unhealthy eating habits, which was also associated with general low self-control ability^27^. On the contrary, enjoyment and health attributes of physical activities were positively correlated in our study. However, the ACT group showed higher positive correlations between enjoyment and healthiness of physical activities compared to the SED group (Fig. 2B), which may make the SED group more susceptible to unhealthy physical activity decisions. In regression models, food and physical activity decisions were solely predicted by the immediate reward-related attributes (taste and enjoyment), not by the long-term benefits-related attribute (health) (Fig. 2C). In group comparisons, somewhat surprisingly, the NW ACT group showed higher taste beta weights compared to the NW SET group (Fig. 2D). While the NW ACT group currently possesses a relatively healthy status regarding BMI and physical activity, their taste-oriented food decision process pattern established during the adolescent period may serve as a risk factor in their later life stages, if they reduce their amount of physical activity, which many youths do as they enter early adulthood. Alternatively, it could be explained according to recent findings by Gauthier and colleagues^28^, who demonstrated that taste intensity/sensitivity can increase following exercise. It is therefore possible, that the higher beta weights for taste in the NW ACT participants are the result of their higher levels of physical activity and could possibly decrease when decreasing their physical activity.

One of our goals was to explore whether food decision-making processes share similar neurocomputational mechanisms with physical activity decision-making processes. Self-control is often defined as the general psychological ability of an individual to control or override one’s impulses or desires for immediate pleasure (e.g., tasty but unhealthy foods, enjoyable but unhealthy activities) to achieve long-term goals (e.g., health benefits). Previous research reported that self-control ability is significantly associated with healthy diet^17^ and exercise^29,30^ habits. However, it is unknown whether self-controlled food (energy intake) decisions are interdependent with self-controlled physical activity decisions. In our study, the proportions of successful self-controlled food decisions were significantly correlated with the proportion of successful self-controlled physical activity decisions, suggesting linked or reciprocal self-control mechanisms of energy intake and expenditure decisions. Our results also demonstrated that the taste-oriented food decisions are positively correlated with the enjoyment-oriented physical activity decisions suggesting common pleasure-seeking decision processes (Fig. 3A). Importantly, the pleasure-oriented food and physical activity decision weights were negatively associated with self-controlled food and physical activity decisions (Fig. 3C, E), and the health-oriented decision weights were positively associated with self-controlled food and physical activity decisions (Fig. 3D, F), suggesting the pleasure-oriented decision process serves as a susceptibility factor while the health-oriented decision process serves as a resilience factor for energy balance self-control decisions. Beyond our hypothetical food and physical activity decisions, we explored whether our key decision-making model variables are linked to ‘actual’ food consumption and physical activity measures. The correlations between food taste and health attribute ratings, *r*_(taste, health),_ were negatively associated with the amount of food consumption at the ad libitum buffet (Fig. 3G)—participants who perceived unhealthy foods as tasty consumed more foods. Similarly, the correlations between activity enjoyment and health attribute ratings, *r*_(enjoyment, health)_, were positively associated with the 2-week physical activity monitor measure. Taken together, our behavioral findings demonstrated that our decision model variables that represent how individuals make food and physical activity decisions significantly explain the actual energy intake and expenditure as well as the self-control outcomes in the experimental setting.

In our fMRI analyses, we found that both food and physical activity decision values are encoded in the vmPFC (Fig. 4A), as we hypothesized. The vmPFC is established as a key brain area for goal value representation^31–33^ for different types of goods such as food, nonfood consumables, clothing, and monetary incentives. In this project, however, we have for the first time demonstrated that the vmPFC also encodes decision values for physical activities. Combined with our computational model, our results suggest that both food and physical activity decisions share common value encoding neural circuits at the time of choice. In group comparisons, compared to the OW/OB group, the NW group showed stronger brain activations in the brain’s cognitive control (IFG), multisensory integration (STG), and motor control (MC) regions during physical activity decisions (Fig. 5B). This result suggests that body weight status can differentially modulate brain circuits that are required to make physical activity decisions. However, contrary to what we hypothesized, we could not find supporting evidence that physical activity level differentially modulates brain circuits during food and physical activity decisions.

This novel neuroimaging study that included both food and physical activity choice tasks demonstrates that these two decision-making processes are interdependent. Importantly, our finding suggests that self-regulated decisions for both food and physical activity utilize similar computational and neurobiological mechanisms, which may provide valuable insights into how to promote healthy food and physical activity decisions for adolescents. For example, an intervention that is designed to de-emphasize the immediate pleasure-oriented decision process and emphasize the long-term health-oriented decision process could be effective to modify both unhealthy energy intake and expenditure behaviors. That is, emphasizing how a person can forgo an immediate fun unhealthy activity or a tasty unhealthy food and instead “be kind to your future self,” could be a helpful framework for health behavior change.

However, there are remaining issues that were not fully addressed in this study. First, in this study, we recruited participants across four categories based on body weight status and physical activity levels to identify specific contributions of each factor with minimal statistical adjustments. However, it is also true that a priori grouping reduces statistical power to detect group differences, especially when the differences are small. We tried to minimize this power issue by only including adolescent males in this project, which of course results in additional, separate limitations in terms of generalizability. We believe that it is important to explain the decision mechanisms in both sexes in the future to check potential differences^34,35^. Also, due to the relatively small sample size, our exploratory group comparison results by weight status and activity levels should be interpreted cautiously as preliminary evidence. Larger samples including children, adolescents, and young adults are required to fully elucidate the developmental decision-making trajectory. Another limitation is the use of an ordinal scale as opposed to a visual analog scale for ratings. This may exclude meaningful variance better captured by a visual analog scale (VAS). We chose an ordinal scale based on our past behavioral and neuroimaging studies^18,20,27,36,37^ using this. Third, another question is how our findings could be generalized to food and physical activity decisions often shared with other persons (e.g., family meals, school lunches, and team sports). It should be noted that some of the physical activity images were group types of activities (i.e., basketball), while others were solitary (i.e., cycling). The social aspect of these activity choices has yet to be fully explored. Lastly, in our study, for compatibility between food and physical activity choices, participants made hypothetical decisions. Thus, while the effort cost can be another important factor for value-based decisions^38–40^, the effort cost was not considered in our study design. However, in real food and activity-related decisions can be substantially different. Future studies are necessary to expand ecological validity and generalizability. In general, the psychological and neurobiological control mechanisms for energy intake and energy expenditure that maintain a healthy and stable energy balance are relatively poorly understood. From an energy balance perspective, preventing weight gain is known to be more effective than treating obesity^9^. Advancing our scientific understanding of how individuals make energy intake and expenditure decisions is imperative for developing interventions that promote lifelong healthy behavioral habit formation in adolescents.

## Methods

### Participants

Thirty-eight adolescent males (14–18 years old; *mean* 15.88 years old; self-reported Tanner Stage III–IV; 28 Caucasian, 7 African American, 2 Asian/Pacific, and 1 Indian/Alaska) completed the behavioral and fMRI tasks. Inclusion criteria included stable body weight (± 5%) over the previous three months and being healthy for physical activity. Participants taking medications known to affect physical activity levels and metabolism were excluded (e.g., thyroid medications, beta-blockers, or other stimulants). No participants reported using antidepressants. Participants had no history of allergies to the food items used in the experiment. The participants were recruited as a part of a larger behavioral study (Clinicaltrials.gov: NCT03157063) that included 6 additional participants who did not participate in (2 participants) or were excluded due to MRI exclusion criteria (dental braces; 2 participants) and technical (1 participant) or attentional issues (> 50% responses misses during MRI tasks; 1 participant). All study protocol was approved by the Children’s Mercy Institutional Review Board. All participants provided informed consent, or in the case of minors, assent in addition to informed consent by a legal guardian. All methods were performed in accordance with relevant guidelines and regulations by our approved protocol.

For experimental purposes, participants were recruited across 2 by 2 categories based on body weight status (normal weight, overweight/obese) and physical activity levels (active, inactive). Based on age- and sex-specific body mass index (BMI; kg/m^2^) percentiles^41^, normal weight (5th percentile to less than the 85th percentile) and overweight/obese (85th percentile to less than 99th percentile) groups were categorized. To avoid severe obesity that is often accompanied by co-morbid conditions, participants above the 99th percentile were not included. Physical activity status was determined through a two-step procedure. During the initial recruitment, 50% active (≥ 60 min/day) and 50% sedentary (inactive) (< 60 min/day) participants were selected based on the self-reported activity level—the minutes spent in active play/exercise (breathing harder or sweating) on a typical day. Next, physical activities during a 14-day free-living period were measured using accelerometry (GT9X, ActiGraph, Pensacola, FL). Participants were instructed to always wear the activity monitor for 14 days, including sleeping and showering. Based on the vector magnitude (VM) average count, a median split was conducted. Those below this median threshold were categorized as sedentary (inactive) and above as active. The final participants consisted of 11 individuals with normal weight and active lifestyle (NW ACT), ten individuals with normal weight and sedentary lifestyle (NW SED), 10 individuals with overweight/obese and active lifestyle (OW/OB ACT), and 7 individuals with overweight/obese and sedentary lifestyle (OW/OB ACT). Table 1 shows demographic characteristics. There was no significant age difference across the four groups, *F*_(3,34)_ = 0.29, *p* = 0.830, *η*_p_^2^ = 0.03.

**Table 1:** Descriptive statistics (*n* = 38).

	All	NW ACT	NW SED	OW/OB ACT	OW/OB SED
*n*	38	11	10	10	7
Age	15.88 (0.93)	16.01 (1.11)	15.89 (1.01)	15.65 (0.93)	16.01(0.60)
BMI percentile	71.98 (25.22)	56.31 (18.71)	50.00 (18.81)	93.99 (4.89)	96.55 (2.03)
VM average count	1866 (423)	2141 (284)	1583 (213)	2177 (316)	1395 (246)

Mean and standard deviations.

Dietician-administered dietary recalls conducted via phone were completed on three randomly selected days (two weekdays, one weekend day) by a registered dietician using a multi-pass approach^42,43^. Participants were asked to recall all foods and drinks consumed in the previous 24 h. The information collected was entered into the Nutrient Data System for Research software (NDSR)^44^ and total daily energy intake was calculated as the average intake over all recalls.

### Procedures

#### Laboratory appetite assessment

Before the 14-day free-living physical activity (activity monitor) and dietary (daily food diary) assessment described above, participants completed one appetite assessment session. The appetite assessment session occurred at 9:00 am following a 12-h dietary fast. Participants were provided a breakfast sandwich, chocolate milk, and 236 mL of water. The amounts of sandwich and chocolate milk varied by participants to match approximately 40% of the measured resting metabolic rate (RMR) that was assessed using a standard protocol^45–47^, while maintaining a macronutrient composition of 50% of kcals from carbohydrates, 30% from fat, and 20% from protein. Participants were asked to eat the entire meal within 15 min. At 3.5 h following the breakfast meal, participants were given access to an ad libitum cheese pizza buffet, served individually in a quiet room. The pizza (58% carbohydrate, 25% fat, 17% protein) was served in 435 kcal portions, cut into six non-uniform pieces, along with 236 mL of water. Participants were instructed that they could eat *as much as they would like* until they were comfortably full. Research associates observed the meal from a window outside of the room and provided a new plate of pizza when the participant began eating the last piece on the plate. The meal was terminated when 5 min passed without the participant eating. Plate weight was measured before and after the meal, and the total kcals consumed were calculated. Participants provided subjective ratings of aspects of palatability following the ad libitum buffet of pizza using a visual analog scale, with 0 being ‘Good’ and 100 being ‘Bad.’ Mean values, respectively, were as follows: Visual appeal, 23.79 (*SD* = 22.61); Smell, 19.74 (*SD* = 20.09); Taste, 16.37 (*SD* = 18.03), Aftertaste, 42.92 (*SD* = 31.42), suggesting participants generally liked the pizza provided. No significant group differences were observed, all *p* values > 0.05.

#### Experimental stimuli

Sixty food images and sixty physical activity images were used (Fig. 1). Food stimuli included 30 healthy food items, such as vegetables, fruits, and milk, and 30 unhealthy food items, such as chocolates, French fries, and bacon. Physical activities were selected based on Metabolic Equivalents (MET)^48^. Physical activity stimuli included 30 moderate or vigorous physical activity items (> 3.0 METs), such as basketball, biking, and hiking, and 30 light physical activity items (< 3.0 METs) such as reading, resting, and watching television.

#### MRI session

On a separate visit following the 14-day free-living assessment period, participants completed a behavioral rating and fMRI decision tasks. Following the common standard of food-related neuroimaging studies^17,18,36^, participants fasted for 4 h to ensure moderate hunger status. Using visual analog scales (VAS)^49^, participants completed subjective ratings of hunger and satiety. Means of hunger and satiety levels on a 100-point scale were 68.26 (*SD* = 20.12) and 24.51 (*SD* = 15.62), respectively, suggesting showed a moderate level of hunger status as intended. No significant group differences were observed, all *p* values > 0.05.

#### Behavioral food and physical activity rating task

Before MRI scans (outside of the scanner), participants completed food and physical activity rating tasks (Fig. 1A). For the food rating task^20,36^, they provided separate taste (very bad–very good) and health (very unhealthy–very healthy) ratings as well as their overall preference (strongly dislike–strongly like) ratings for 60 food items. Similarly, for the physical activity rating task, they provided separate enjoyment (very unenjoyable–very enjoyable) and health (very unhealthy–very healthy) ratings as well as their overall preference (strongly dislike–strongly like) ratings for 60 physical activity items. This also ensured that images would be recognizable when presented with them in the MRI.

#### fMRI food and physical activity decision task

Participants completed a series of choices for each food and physical activity item in two different types of condition blocks (‘food’ and ‘physical activity’) that were randomly presented (Fig. 1B). In the food choice condition^20,36^, participants made decisions about whether they ‘*want to eat’* the food shown. In the activity choice condition, participants made decisions about whether they ‘*want to do’* the physical activity shown on the screen. Even though they were hypothetical choices, participants were encouraged to make their decisions as real as possible.

### MRI data acquisition and preprocessing

Anatomical (1 mm isotropic voxel) and functional (TR = 2 s; TE = 25 ms; FA = 90°; 3 mm isotropic voxel) MRI data were acquired using a Siemens 3 T Skyra scanner (Siemens Medical Systems, Germany) with a 32-channel head coil at the Hoglund Biomedical Imaging Center of the University of Kansas Medical Center. The AFNI package^50^ was used for preprocessing and statistical analyses of fMRI data. All participants showed less than 3 voxels of head motion (< 9 mm). We applied slice-time correction, motion correction, spike correction (3dDespike), spatial resampling (3 × 3 × 3 mm) and normalization to the standard Talairach template, Gaussian spatial smoothing (FWHM: 6 mm), and intensity normalization (each voxel’s mean was set to 100).

### Statistical analyses of fMRI data

We performed a general linear model (GLMs) that allows for first-order autoregression (AR1) and included six motion parameters, constants, and time trends for each run as regressors-of-non-interest. We performed a two-stage mixed-effects analysis in which the regression coefficients for each condition of interest were tested across participants via *t* tests (two-tailed tests). Multiple comparison corrections were implemented at the cluster level using Monte Carlo simulations with the 3dClustSim program (http://afni.nimh.nih.gov). The whole-brain level statistical inferences were made at a corrected threshold of *p* < 0.05 by imposing a *p* < 0.001 and a minimum cluster extent of 22 voxels. For pre-determined regions of interest, we used small volume corrections (SVC) at the cluster level (*p* < 0.001 and extent threshold of 5 voxels for vmPFC and 4 voxels for striatum). The anatomically defined vmPFC mask consisted of medial orbital gyrus, rectal gyrus, and olfactory cortex masks of AFNI’s standard anatomical brain^50^. The anatomically defined striatum mask consisted of putamen, caudate, and lentiform nucleus. Activations are reported using Talairach coordinates^51^.

#### General linear model (GLM)

Using a model-based fMRI data analysis approach^21,22^, we fitted the GLM on all choice trials to identify brain areas that encode food and physical activity decision values at the time of the choice. The model included (1) an indicator function (1 for events, 0 otherwise) for the food choice period (with an RT duration), (2) the indicator function for the food choice period multiplied by the food decision values measured through behavioral responses (‘strong no’, ‘no’, ‘yes’, ‘strong yes’), (3) an indicator function for the physical activity choice period, and (4) the indicator function for the physical activity choice period multiplied by the physical activity decision values measured through behavioral responses (‘strong no’, ‘no’, ‘yes’, ‘strong yes’).

We used AFNI’s 3dDeconvolve program with the AM2 amplitude modulator option and the two parametric regressors included in this GLM allowed us to identify brain areas that encode the food and physical activity decision variables.

## Supplementary Information

Supplementary Tables.

## Data Availability

The experimental paradigm and stimulus sets are available at https://osf.io/7t4wr/. The datasets generated during and/or analyzed during the current study are available from the corresponding author upon reasonable request.
