# Supplementary material for: Neurocomputational mechanisms of food and physical activity decision-making in male adolescents
Source: Sci Rep. 2023 Apr 15;13:6145. doi: 10.1038/s41598-023-32823-x (PMC10105706; doi:10.1038/s41598-023-32823-x)
Supplement: Supplementary file 1 — Supplementary Tables. [file 41598_2023_32823_MOESM1_ESM.pdf]

Table S1. Descriptive statistics of behavioral ratings (*n* = 38)

**Food ratings**	*Mean*	*SD*	**Physical activity ratings**	*Mean*	*SD*
Unhealthy - taste	3.08	.31	Sedentary - enjoyment	2.60	.26
Unhealthy - health	1.83	.21	Sedentary - health	2.39	.28
Unhealthy - preference	3.67	.46	Sedentary - preference	3.08	.41
Healthy - taste	2.91	.29	Active - enjoyment	2.95	.30
Healthy - health	3.41	.25	Active - health	3.42	.24
Healthy - preference	3.39	.46	Active - preference	3.58	.49

Food taste and healthy attribute ratings and activity enjoyment and health attribute ratings were acquired through the 4-point scale measures. Food preference and physical activity preference ratings were acquired through the 5-point scale measures.

Table S2. Brain regions correlated with food and activity decision values at the time of choices.

Region	L/R	Talairach (peak)			*t*	cluster size
		x	y	z		
**Food decision value**						
Ventromedial Prefrontal Cortex (vmPFC)	R	11	41	-4	4.18 ^{svc}	6
Anterior Insula	R	29	20	-1	-5.23	24
**Activity decision value**						
Ventromedial Prefrontal Cortex (vmPFC)	L/R	-10	35	-1	4.28	8
Anterior Insula / Putamen	R	23	5	11	-4.64	30
Middle Frontal Gyrus	R	44	26	26	-5.13	130
Inferior Parietal Lobule	L	-43	-34	41	-4.11	32
Fusiform Gyrus	R	32	-40	-16	-4.92	24
Superior Parietal Lobule	L	-28	-61	47	-5.69	165
	R	23	-58	41	-5.94	250
Inferior Occipital Gyrus	R	50	-58	-10	-5.18	30
Middle Occipital Gyrus	L	-37	-79	2	-4.96	45
	R	35	-76	5	-4.30	43
**Food – Activity decision value**						
Precentral Gyrus	R	47	-10	47	6.08	24
Superior Parietal Lobule / Precuneus	R	11	-49	53	4.84	28

$p < .05$ with whole brain cluster size correction (height threshold $t = 3.56$, $p < .001$; extent threshold $k = 22$ voxels); ^{svc}, $p < .05$ with small volume correction (height threshold $t = 3.56$, $p < .001$; extent threshold $k = 5$ voxels for vmPFC).

Table S3. Brain regions activated at the time of food and activity choices.

Region	L/R	Talairach (peak)			*t*	cluster size
		x	y	z		
**Food decision > baseline**						
Orbito-Frontal Cortex (OFC)	L	-19	41	-7	10.36	382
	R	23	50	-10	8.56	409
ventral Striatum / Caudate	L	-22	2	-7	7.41	480
	R	20	-1	-7	6.99	444
Superior/Middle Frontal Gyrus	L	-25	44	32	8.72	335
	R	41	44	8	6.72	247
Middle Cingulate Gyrus / Pre-Supplementary Motor Area (Pre-SMA)	L/R	2	23	35	6.25	392
Anterior Insula	L	-34	11	2	6.30	136
	R	47	11	2	5.78	49
Thalamus	L/R	-1	-10	14	8.12	262
Posterior Cingulate Gyrus	L/R	-1	-37	26	8.38	56
Supra-Marginal Gyrus / Inferior Parietal Lobule	L	-37	-40	32	6.75	215
	R	44	-58	41	6.45	245
Superior Temporal Gyrus	L	-58	-22	20	4.34	26
	R	59	-25	23	5.18	40
Cuneus / Precuneus	L/R	2	-76	35	9.47	480
Fusiform Gyrus / Inferior Temporal Gyrus	L	-55	-55	-19	8.50	151
	R	53	-64	-19	8.26	279
Lingual Gyrus	L/R	-7	-58	-7	9.42	285
**Activity decision > baseline**						
Orbito-Frontal Cortex (OFC)	L	-31	50	-4	7.90	447
	R	23	47	-10	9.32	452
ventral Striatum / Caudate	L	-19	-1	-4	7.10	375
	R	26	2	-4	7.32	217
Superior / Middle Frontal Gyrus	L	-28	47	32	7.69	349
	R	41	35	29	5.84	268
Middle Cingulate Gyrus / Pre-Supplementary Motor Area (Pre-SMA)	L/R	-1	20	50	6.70	299
Anterior Insula / Inferior Frontal Gyrus	L	-46	20	-1	5.67	86
Thalamus	L/R	-1	-13	14	8.51	266
Posterior Cingulate Gyrus	L/R	-1	-40	23	7.75	93
Inferior / Superior Parietal Lobule	L	-40	-61	44	6.76	120
	R	44	-58	41	6.05	274
Cuneus / Precuneus	L/R	2	-82	29	8.96	460
Fusiform Gyrus / Inferior Temporal Gyrus	L	-43	-52	-25	8.24	245
	R	50	-49	-25	10.43	300
Lingual Gyrus / Parahippocampal Gyrus	L/R	-4	-37	5	9.24	285
**Food – Activity decision**						
Orbito-Frontal Cortex (OFC)	L	-25	35	-1	6.34	27
Ventromedial Prefrontal Cortex (vmPFC)	L/R	-7	38	-7	-5.12	66
Fusiform Gyrus / Parahippocampal Gyrus	R	41	-31	-16	-5.04	46
Middle Occipital Gyrus	L	-46	-76	2	-6.14	192
	R	47	-76	-4	-5.68	82

$p < .05$ with whole brain cluster size correction (height threshold $t = 3.56$, $p < .001$; extent threshold $k = 22$ voxels)

Table S4. Brain regions activated differently by group membership at the time of food and activity choices.

Region	L/R	Talairach (peak)			*z*	cluster size
		x	y	z		
**Weight Status by Activity Status interaction effect**						
Pre-Supplementary Motor Area (Pre-SMA)	L/R	2	17	44	4.11	30
Cerebellum	L/R	-7	-82	-22	4.37	22
**NW > OW/OB group: Food decision**						
None						
**NW > OW/OB group: Activity decision**						
Inferior Frontal Gyrus / Anterior Insula	R	44	29	11	3.90	73
Precentral / Postcentral Gyrus	L	-31	-25	62	3.61	25
	R	47	-25	50	4.03	51
Superior Temporal Gyrus	L	-61	-43	14	4.34	77
	R	47	-40	-1	4.38	56
Angular Gyrus	R	41	-64	26	4.38	72
**Active > Sedentary group: Food decision**						
None						
**Active > Sedentary group: Activity decision**						
None						

$p < .05$ with whole brain cluster size correction (height threshold z = 3.28, $p < .001$; extent threshold $k = 22$ voxels).
